# The Role of Autologous and Allogeneic Stem Cell Transplantation in Follicular Lymphoma in The New Drugs Era

**DOI:** 10.4084/MJHID.2016.045

**Published:** 2016-09-01

**Authors:** Francesco Maura, Lucia Farina, Paolo Corradini

**Affiliations:** 1 Division of Hematology and Bone Marrow Transplant, Fondazione IRCCS Istituto Nazionale dei Tumori, Via Venezian 1, Milan, Italy; 2 Department of Oncology and Hemato-oncology, University of Milan, Milan, Italy

## Abstract

Follicular lymphoma (FL) is the second most common histotype of non-Hodgkin’s lymphoma, and it is generally characterized by a heterogeneous clinical course. Despite recent therapeutic and diagnostic improvements, a significant fraction of FL patients still relapsed. In younger and/or fit FL relapsed patients bone marrow transplant (BMT) has represented the main salvage therapy for many years. Thanks to the ability of high-dose chemotherapy to overcome the lymphoma resistance and refractoriness, autologous stem cell transplantation (ASCT) can achieve a high complete remission rate (CR) and favorable outcome regarding progression-free survival (PFS) and overall survival (OS). Allogeneic stem cell transplantation (alloSCT) combines the high dose chemotherapy effect together with the immune reaction of the donor immune system against lymphoma, the so-called ‘graft versus lymphoma’ (GVL) effect. Considering the generally higher transplant-related mortality (TRM), alloSCT is mostly indicated for FL relapsed after ASCT. During the last years, there have been a great spread of novel effective and feasible drugs Although these and future novel drugs will probably change our current approach to FL, the OS post-BMT (ASCT and alloSCT) has never been reproduced by any novel combination. In this scenario, it is important to correctly evaluate the disease status, the relapse risk and the comorbidity profile of the relapsed FL patients in order to provide the best salvage therapy and eventually transplant consolidation.

## Introduction

Follicular lymphoma (FL) is the second most common histotype of non-Hodgkin’s lymphoma with an incidence of approximately 15.000 new cases/year in the United States.^1^ The incidence increases with age, with the median age at diagnosis being 60 years. FL clinical course is generally heterogeneous with progression-free survival (PFS) ranging from 71% to 35% at 10 years, according to the Follicular Lymphoma Prognostic Index Score (FLIPI),^2^ and 91% to 51 % at 3-year, according to with Follicular Lymphoma Prognostic Index Score-2 (FLIPI-2).^3^ During the last years, the FL clinical management has been progressively improved thanks to the use of more accurate and sensible diagnostic technics such as the Positron Emission Tomography (PET) and the Minimal Residual Disease (MRD) monitoring^4^–^11^ and the introduction of novel effective agents.^12^ Anti-CD20 humanized antibody Rituximab led to one of the most important changing in FL clinical practice, and probably still represents the major improvement in FL therapy and outcome in the last 20 years.^13^–^15^ Indeed, different studies show how the combination of rituximab and standard chemotherapy (i.e, CHOP) can significantly improve the FL prognosis and survival compared to standard chemotherapy alone. Despite all these advances, a significant proportion of FL patients still experiences an early or late relapse.

Bone marrow transplant (BMT) has been widely investigated to achieve a better response and improve the survival in FL.^16^–^18^ Autologous stem cell transplantation (ASCT) has represented one of the main treatment for relapsed FL thanks to the ability of high-dose chemotherapy to overcome the lymphoma resistance and refractoriness. Allogeneic stem cell transplantation (alloSCT) combines the high dose chemotherapy effect together with the immune reaction of the donor immune system against lymphoma, the so-called ‘graft versus lymphoma’ (GVL) effect. Although the BMT anti-lymphoma activity is generally superior to standard chemotherapy, this approach is limited to young and fit patients due to the transplant-related toxicity and mortality (TRM). In the last years, thanks to the novel less toxic conditioning regimens available and to the significant improvement in supportive cares, the proportion of FL patients potentially eligible to ASCT and/or alloSCT has progressively expanded. Today these procedures are feasible up till the age of 65–70 years.^16^, ^17^

In this review we examine the current state of the art of FL treatment, focusing in particular on the role of BMT and its indications.

## Transplant in the First Line: End of the Story?

In the pre-rituximab era, three different main trials investigated the role of intense chemotherapy with final ASCT consolidation as first-line therapy for FL patients.^19^–^21^ Although this approach showed a significant progression-free survival (PFS) improvement, the advantage was abolished by the high incidence of therapy-related malignancies seen in the ASCT arms.^22^–^24^ The Gruppo Italiano Trapianto di Midollo Osseo (GITMO) investigated the combination of rituximab with high-dose sequential (HDS) chemotherapy regimens, including ASCT as final consolidation, showing a significant improvement in terms of PFS and overall survival (OS).^25^ However, the first randomized trial conducted by GITMO and Intergruppo Italiano Linfomi (IIL) failed to demonstrated any survival advantage of R-CHOP followed by ASCT compared to R-CHOP alone in high-risk FL patients.^7^ Enrolling a total of 136, patients this study represents the main clinical trial that investigated the role of ASCT in the first line FL in rituximab era. After a median follow-up of 51 months, the ASCT arm showed a clear 4-year event-free survival (EFS) advantage (62% versus 28%); however, no difference was seen in OS (81% versus 80%), confirming what was previously described in trials without the anti-CD20 monoclonal antibody. The final results of this trial were particularly interesting considering the MRD monitoring data. A significantly higher rate of molecular remissions (MRs) was achieved in the ASCT arm compared with the conventional chemotherapy (80% versus 44%, respectively), with MRD status being the strongest predictor of outcome. Importantly, patients randomized to the non-transplant arm that achieved MRD negativity did not show any difference in outcome compared to MRD-negative patients who underwent ASCT.^7^ These data suggest that MRD negativity should be considered as one of the main end points in FL therapy.

A further improvement in PFS post R-CHOP was recently obtained by the introduction of rituximab maintenance, as demonstrated by the PRIMA trial.^26^ The last update showed a PFS at 6-year of 59% in maintenance vs. 42.7% of the non-maintenance arm.^27^ These results demonstrate that standard immune-chemotherapy regimens may achieve a long remission in more than half FL patients. A sub-analysis of the PRIMA study also showed that PET-positive patients had a significantly inferior PFS at 42 months compared to those who became PET negative (32.9 versus 70.7%). The risk of death was also increased in PET-positive patients.^28^ In this perspective, the introduction of the PET scan into the clinical practice may further improve the disease response evaluation, because it may be able to detect patients with refractory disease or with an early complete remission.^5^,^9^–^11^,^29^ An ongoing prospective trial of the Fondazione Italiana Linfomi (FIL) group is evaluating whether the disease assessment by combination of MRD monitoring and PET scan after rituximab-based induction therapy can identify FL patients who need an intensification or, conversely, those that may avoid rituximab maintenance therapy (EUDRACT NUMBER: 2012-003170-60).

Finally, the incorporation of novel agents such as bendamustine and lenalidomide in the first line treatment showed promising results that may produce further potential improvement in term of efficacy and toxicity among all FL.^30^–^34^

In conclusion, considering the absence of survival benefit of ASCT consolidation and the continuous improvements in non-transplant approaches, ASCT should be avoided in the first line therapy for FL patients.

## Relapsed Follicular Lymphoma Patients: New Drugs vs. High Dose Chemotherapy

**Figure 1:**
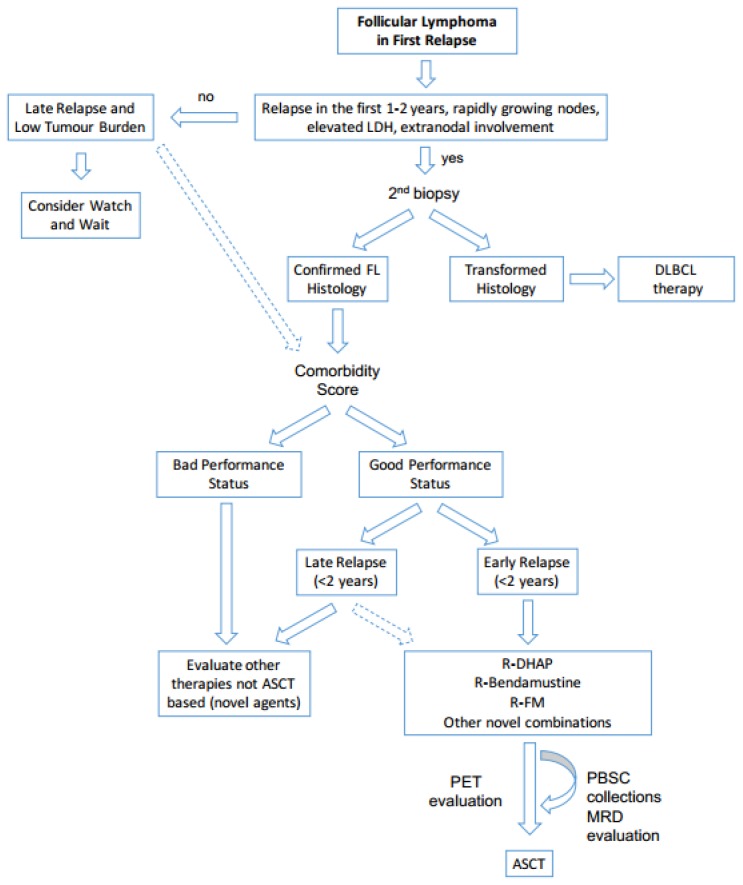
Follicular Lymphoma in First Relapse.

Although the significant therapeutic advances achieved over the last years, approximately 40% of all FL patients relapse in a different way and at different time after the first line.^12^, ^18^ A recent study showed a dismal clinical outcome among FL patients relapsed in the first year after R-CHOP with a 5-year OS rate of 34% (95% CI, 19% to 60%), confirming the early relapse as an indirect parameter of refractoriness and poor outcome.^35^ Based on these data, it is crucial to provide the best salvage therapy for these high-risk and early relapsed FL patients. Conversely, the FL patients who relapse after more than two years are associated with a very favorable survival with a 5-year OS of 94%,^12^,^35^,^36^ and the indication for ASCT as salvage treatment in this subgroup is not clear.^37^ In fact, the treatment choice (ASCT versus other less intensive regimens) should always be based on patient symptoms, disease burden such as the presence of bulky disease and/or extranodal involvement, and/or signs of rapidly progressive lymphoma. For patients relapsed after 2 years, intense salvage therapies including ASCT are usually delayed. In case, localized relapse may be managed by the radiotherapy alone and/or monoclonal antibodies postponing more intense approaches. For this purpose, a careful staging is mandatory in all relapsed FL. Figure 1 summarizes our current approach to FL in first relapse after immunochemotherapy.

FL can transform into an aggressive lymphoma at different times.^38^ For this reason, all early relapsed FL and/or those with rapidly growing nodes, elevated LDH and/or significant extra-nodal involvement, should be investigated to rule out a potential transformation in diffuse large B-cell lymphoma (DLBCL). In this relapsed setting, the differential diagnosis is mandatory, because the biological and clinical behavior of transformed FL is similar to the one of the DLBCL, thus requiring intensive chemotherapy and ASCT.^39^

Before starting any treatment, an important element to consider other than histologic transformation is the patient comorbidity status. In fact, intense salvage schemes and ASCT consolidation should be avoided in old (≥65–70 years) and/or frail patients, considering the risk of significant toxicity and complications. Nevertheless, the definition of elderly or frail is not unique, as addressed in the last paragraph of this review.

In the case of young and fit early relapsed FL patients, high dose chemotherapy approaches plus ASCT consolidation showed the best results in terms of OS and PFS (Table 1).^24^,^25^,^40^–^46^ This outcome was further improved by adding rituximab into intense salvage regimens. A GITMO retrospective analysis reported a 5-year EFS and OS of 65% and 80%, respectively, among refractory and early relapsed FL treated with Rituximab in combination with high-dose sequential therapy (R-HDS).^25^ Similar results were achieved in late relapsed FL cases with a 5-year EFS and OS of 71% and 83%. Overall these data showed a significant improvement compared to the HDS regimens without rituximab (5-year EFS and OS of 23% and 41%, respectively among early relapsed and 33% and 65% respectively among late relapsed FL patients). In this retrospective study authors also included rituximab naïve relapsed FL patients, and this may partially explain excellent results. Nevertheless, in a subanalysis including together all DLBCL and FL previously treated with rituximab, the 5-year EFS, and OS were 44% and 56%, respectively. Considering previously published studies (Table 1) including relapsed FL patients, the 5-year PFS after high dose regimens and ASCT consolidation is approximately 40–50%. Furthermore, it is well known that the addition of anti-CD20 monoclonal antibodies is able to improve the MRD-negative peripheral stem cell harvests and, consequently, the survival and the rate of MRs after ASCT.^7^,^8^,^47^–^50^

In the last 10 years, different alternative less toxic approaches were tested among relapsed/refractory FL. Bendamustine represents one of the most important agent with a reported overall response rate (ORR) of 81% and a median PFS of approximately 8 months as a single agent in relapsed FL patients.^51^ Interestingly, the combination of Rituximab plus Bendamustine in the first line did not show any inferiority compared to R-CHOP, confirming the high efficacy of this agent and its strong synergistic activity with rituximab.^30^,^32^,^33^,^51^–^54^ Fludarabine-containing regimens still remain a valid option, although their use should be carefully evaluated considering the high rate of early and late toxic adverse events.^55^ An effective agent in relapsed FL is represented by radio-immunotherapy (i.e., Zevalin®) that should be considered preferentially in non-bulky relapse with a bone marrow infiltration <20%.^56^,^57^

Most recently, several novel molecules have shown an interesting activity in relapsed/refractory FL patients (Table 2).^12^ Particularly, lenalidomide, pidilizumab, and idelalisib demonstrated a strong activity and an acceptable toxic profile also when combined with rituximab.^58^–^62^ In addition, few novel and potentially more active anti-CD20 monoclonal antibodies (GA-101 and Ofatumumab) are currently under investigation,^63^–^65^ as well as novel conjugated monoclonal antibodies (i.e., Polatuzumab).^66^ Overall, these new molecules are progressively bringing significant changes in relapsed FL management, and they may improve the outcome in the next future. A challenging issue is how to integrate these drugs into the therapeutic strategy and how to combine them with chemotherapy. In fact, although these molecules are not defined as “conventional chemotherapy”, they may be responsible for very important and unknown toxicities. As a matter of fact, two different trials testing the combination of novel agents [Pi3K inhibitor (Idelalisib) + Immunomodulatory agent (Lenalidomide) and Pi3K inhibitor (Idelalisib) + Syk Inhibitor (Entospletinib)] were recently interrupted for unacceptable toxicity and adverse event incidence.^67^,^68^ In the future, it is conceivable that new non-chemotherapic drugs will change the salvage therapy paradigm in FL completely. However, although a significant fraction of relapsed and heavily pretreated FL patients achieved disease reduction when treated with these novel agents, the median PFS has never reached in most of the experiences the survival reported in ASCT studies, so far (Table 1–2). Moreover, ASCT is well known to induce long-term remission in approximately 40–50% of patients. For these reasons, we believe that ASCT still represents the current standard of care of young and fit FL patients in first and early relapse.

## Conditioning Regimens for Autologous Stem Cell Transplantation

The conditioning regimen represents one of the most important therapeutic factors to obtain the best response after ASCT. The most used conditioning regimens are based on high dose chemotherapy, TBI–containing regimen and more recently the combination of chemotherapy and radio-immunotherapy. Although there are not any significant data for a specific conditioning regimen, the use of total-body irradiation (TBI) is associated with a higher risk of secondary malignancy after transplant compared to the chemotherapy-based conditioning regimen.^22^,^23^ Specifically, in the setting of ASCT, the relative risk of therapy-related myelodysplasias or leukemia development was four times more in the TBI-containing group compared to others.^23^ For this reason, this approach has been progressively abandoned. Conversely, radio-immunotherapy, and in particular 90Y-ibritumomab tiuxetan (Zevalin®) represents a valid option not only for the peculiar efficacy that exploits the radiosensitivity of lymphoma cells but also for its safety and acceptable toxic profile.^56^,^57^,^69^ This agent was included in myeloablative conditioning to increase the anti-lymphoma activity of ASCT, in particular in high tumor burden and/or refractory patients at the time of transplant.^70^,^71^ Although the early results are promising, it is not clear what is the real advantage of this combination.

In order to extend the number of patients eligible to ASCT, considering the frequent old age of FL patients, differently less toxic conditioning regimens have been explored in the last years. High dose 90Y-ibritumomab tiuxetan (0.8–1.2 mCi/kg) consolidation after five high dose chemotherapy courses showed promising results among frail and/or elderly series of relapsed and naïve B-lymphomas patients.^72^,^73^ Although the early toxicity profile and the outcome was excellent; the updated results showed an 8-year cumulative incidence of the secondary myelodysplastic syndrome of 9.4%, suggesting an increased risk compared with what was previously reported among younger patients receiving high-dose therapy and autograft. On the other hand, a high incidence of myeloid cancers was not clearly observed when Zevalin® was administered at the standard dose.

Despite the high efficacy of ASCT salvage programs in FL, a significant fraction of patients still relapse. To further improve the efficacy of ASCT, different post-transplant therapeutic approaches have been explored. Rituximab post-transplant maintenance is probably the most important one.^36^,^47^,^48^,^74^,^75^ However, conversely to what reported in the first line, there is no sufficient evidence supporting the use of rituximab maintenance in all FL patients achieving a response after autologous ASCT. A potential setting may be represented by FL patients that do not achieve MRD negativity after transplant. In a small report, it was shown that the percentage of MRs might be increased by a short rituximab consolidation. Further prospective validations are needed to confirm these data.^36^,^47^,^48^,^74^,^75^

## Allogeneic Stem Cell Transplantation

AlloSCT represents an effective treatment for relapsed and refractory FL with 5-years OS ranging from 50–80%.^16^ Historical studies that compared PFS curves of patients who received ASCT or alloSCT reported a statistically significant advantage for allografted patients. However, the lower relapse risk with alloSCT was offset by the higher TRM compared with ASCT, leading to similar 5-year OS rates of 51%–62%.^76^–^78^ For this reason, the best choice in first relapse patients is still represented by ASCT (Figure 1), as previously mentioned. Taking into account that about 30–50% of autografted patients relapse afterward, alloSCT represents an effective salvage strategy that should always be considered in these patients. Retrospective data on myeloablative alloSCT in FL showed a high and quite unacceptable toxicity and TRM incidence.^76^–^78^ For this reason, in the past, alloSCT was generally avoided in the majority of relapsed/refractory FL patients that were usually excluded from myeloablative transplant programs due to the age older than 50–55 years. In order to extend the fraction of eligible FL patients and to reduce the TRM, different less toxic and more feasible conditioning regimens and transplant strategies have been explored. In the last twenty years, the most important change in transplant approach has been represented by the introduction of reduced intensity conditioning (RIC) regimens which are associated with a reduced TRM without a loss of efficacy provided by the GVL effect.^16^,^17^,^79^ Thanks to these qualities, RIC alloSCT extended the application of allotransplant to a larger rate of FL patients that were historically considered ineligible, including also patients older than 60 years old. The first MD Anderson RIC allogeneic SCT study in indolent B-cell lymphomas showed that a conditioning regimen containing fludarabine and cyclophosphamide could provide stable engraftment of donor cells with a low TRM rate.^79^ This study showed a 2-years OS of 80% that was impressive considering that the patients were highly pre-treated and refractory. A recent update of this study confirmed this excellent outcome with an estimated EFS and OS of 83% and 85%, respectively, after a median follow-up of 60 months.^80^ Subsequently, other studies confirmed RIC efficacy and feasibility in relapsed/refractory FL patients, suggesting the existence of a strong immune-mediated antitumor activity (Table 3).^81^–^92^ Indeed, the GVL effect emerged to be superior in patients affected by FL compared to those affected by other lymphomas such as DLBCL.^81^ As a matter of fact, FL represents the only hematological disorders where alloSCT is not contraindicated in case of active disease before transplant. Indeed, several studies show that alloSCT can achieve long-term remissions and a potential disease eradication in approximately 40–50% of FL patients with refractory and active disease before transplant.^93^ This high alloSCT immune activity was further confirmed by the additional evidence of clinical and molecular responses after the withdrawal of immunosuppressive therapy or donor lymphocyte infusions.^80^

A recent multicentre retrospective study included 183 relapsed/refractory after ASCT FL patients who received alloSCT RIC.^88^ The 5-year PFS and OS were 47.7% and 51.1%, respectively, with an acute GVHD cumulative incidence of 45% at 100 days and a 2-year chronic GVHD cumulative incidence of 51.2%. After a median follow-up of 58.8 months (range 3–159), the overall TRM was 24%. Overall, these data confirmed the efficacy of alloSCT among post ASCT relapsed FL patients and, it underlined that TRM and GVHD remain a big issue that affects approximately a quarter of all FL receiving a RIC alloSCT.

Over the years, the TRM incidence decreased thanks to a better understanding of the GVHD and a more accurate diagnosis and management of infectious complications. In particular, the T-cell depletion has been shown to decrease GVHD-related toxicity and mortality, without affecting the relapse risk after transplant.^17^ Unfortunately, despite the introduction of these new transplant strategies, acute and chronic GVHD still represent an important issue and thus novel integrated drugs are currently being explored in alloSCT setting. MD Anderson Cancer Centre showed an interesting and favorable outcome of the inclusion of anti-CD20 monoclonal antibody before and after transplant.^80^,^94^ Other clinical trials are currently investigating how rituximab and novel monoclonal antibodies may improve the immunosuppression without affecting the GVL activity.

Historically, a good HLA-identical donor (sibling or unrelated) is available only for 50–60% of patients, thus representing one of the main alloSCT limitations. Recently, novel transplant approaches have been developed to use an alternative donor in lymphoma patients. Haploidentical donors have been used for many years, mostly after extensive T-cell depletion of peripheral stem cells, to avoid the risk of GVHD. However, this kind of regimen was affected by an unacceptable infectious toxicity and a low feasibility that significantly limited their extensive use in the clinical practice.^95^ Recently, the introduction of T cell-repleted haploidentical transplant using post-transplant cyclophosphamide has shown an interesting combination of low toxicity and low GVHD incidence compared to the historical haploidentical T-depleted SCT.^95^–^101^ Furthermore, a large retrospective study recently suggested that there were no significant differences regarding GVHD incidence and toxic events between haploidentical T-cell repleted SCT and HLA-matched unrelated donors.^102^ This new approach may guarantee the availability of a potential donor for all FL patients, extending the proportion of refractory and relapsed FL patients potentially eligible to alloSCT.

As well as in ASCT setting, significant efforts were performed in order to improve the anti-lymphoma activity of conditioning regimens without affecting neither the immunosuppression nor the GVL process. Excluding the previously cited rituximab-based RIC regimens, two main drugs have been successfully included in conditioning regimens: bendamustine and Zevalin®. Bendamustine combines the alkylating activity of the mustard group with the antimetabolite activity of the analog purine structure, and for this reason, it provides both a potential antitumor and immunosuppression activity. Considering these peculiar characteristics, MD Anderson tested a new RIC bendamustine-based for indolent lymphomas. The results are impressive, with 2-year OS and PFS rate of 90% and 75%, respectively, after a median follow-up of 26 months (range, 6–50 months).^103^ Interestingly, the incidence of acute grade II–IV GVHD was 11%, and the 2-year rate of extensive chronic GVHD was 26%. Ongoing different clinical trials are exploring novel bendamustine alloSCT conditioning regimens to evaluate the major potential benefit and low toxicity effect compared to the historical fludarabine-based regimens. Zevalin®, as mentioned before, represents a well-known active therapy in relapsed FL. Similarly to ASCT, some groups investigated the role of radio-immunotherapy into the alloSCT conditioning regimens.^94^ An interesting combination of (90)Y-ibritumomab tiuxetan (0.4 mCi/kg) and one of the most used fludarabine-based RIC regimens, ((90)YFC) showed a cumulative incidence of grade II–IV acute GVHD at 100 days of 17% (±11%) and chronic GVHD at 12 months of 63% (±19%). The 2-year non-relapse mortality was 18% (±12%), and 2-year OS, and PFS were 83% (±11%) and 74% (±13%), respectively. Although this novel conditioning regimen looks promising, It still needs to be established whether inclusion of radioimmunotherapy in the alloSCT program can significantly improve the final post alloSCT outcome.

The FL biology knowledge is constantly improving, and novel pathogenetic mechanisms and biological subgroups have been described thanks to innovative genomic approaches.^38^ In the next future, this improvement will provide new potential therapeutic targets and may help discover distinct molecular features possibly associated with poorer or better outcome after alloSCT. For example, the recent study of Kotsiou E. *et al.* represents an early and very interesting example of this. Authors reported a favorable post alloSCT outcome associated with TNFRSF14 aberrations that affect 40% of all FL patients.^104^ This is the first demonstration of how a specific tumor genetic lesion may affect the capacity of tumor cells to stimulate allogeneic T-cell immune responses, generating wider consequences for adoptive immunotherapy strategies.

**Figure 2:**
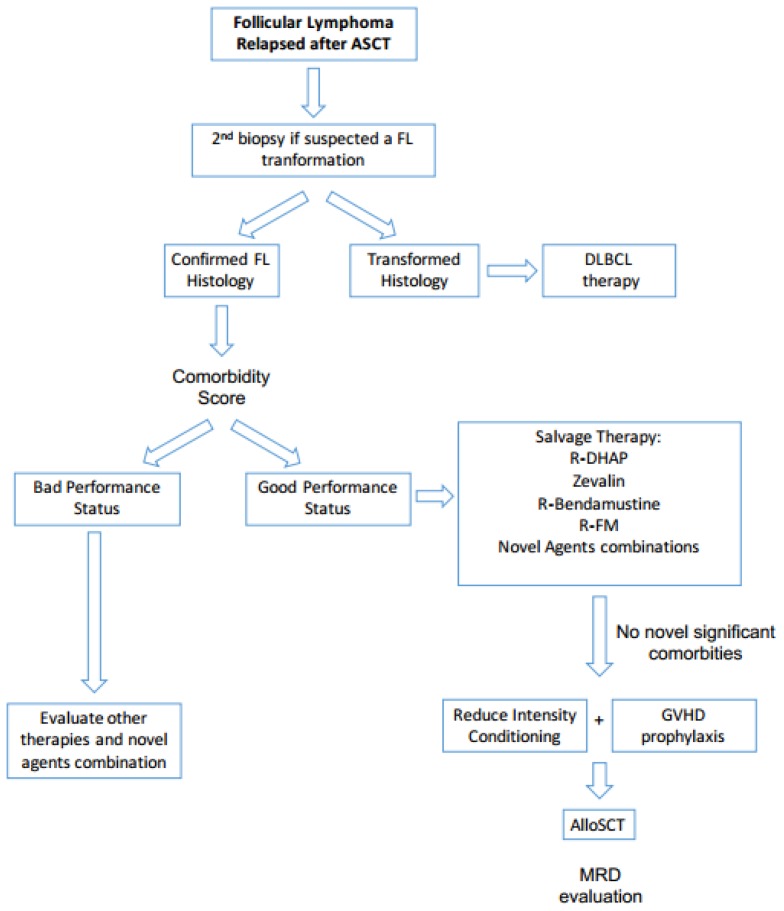
Follicular Lymphoma Relapsed after ASCT.

Considering all these data, in our current clinical practice we candidate all eligible FL patients who relapse after ASCT to alloSCT (Figure 2).

## Transplant: is Always an Age Issue?

Overall, alloSCT and ASCT anti-lymphoma efficacy are well established among young/fit relapsed FL patients. The OS and PFS results after these approaches have never been reached by any novel new drugs. Nevertheless, the long-term outcome of transplanted patients may be affected by late toxic events, especially in the alloSCT setting. In this scenario, a correct and multidisciplinary evaluation of the performance status and comorbidities of all relapsed FL patients should always be provided before the transplant, as well as a careful long-term follow-up after SCT. During the last years, several studies have highlighted that the sole age is not enough to exclude patients from more intense salvage approaches such as BMT. Different comorbidity scores have been proposed to stratify the risk of toxic and adverse events related to high-dose chemotherapy regimens.^105^–^110^ Overall, these scores showed how relapsed FL patients older than 65 years but with a low number of comorbidities may have a significant benefit from intensive chemotherapy regimens without an excess of toxicity. This is possibly due to RIC regimens, better supportive care and less toxic pre-transplant treatments both in ASCT and in alloSCT setting. The most used score in the alloSCT setting is represented by the hematopoietic cell transplantation comorbidity score (HCT-CI).^106^ This score is able to predict the TRM of patients undergoing an alloSCT, regardless of disease status. Recently, the HCT-CI was updated including age through different parameters.^109^ The age threshold was 40 years old, confirming that among the over 40 years old patients, age was not sufficient to assess and stratify the risk of toxicity. Having more than half of FL patients older than 60 years old, this score should be applied in all the patients undergoing an alloSCT.

The importance of patient comorbidity status straightened the role of timing in transplant decision-making. The transplant has not to be considered as a “magic wand” able to eradicate lymphoma cells in every time and every condition. The transplant outcome depends on many factors: delaying alloSCT or ASCT to subsequent relapses exposes patients to other salvage therapies, other potential comorbidities, as well as problems in donor availability. In conclusion, if we have an advanced early relapsed FL patient with a low comorbidity index, ASCT should not be delayed and alloSCT should be performed as soon as the patient achieves the best response after ASCT relapse.

## Conclusion

The continuous introduction of novel and effective therapies is rapidly changing the traditional approaches to different hematological cancers. As well as other lymphoproliferative disease, also FL is strongly involved by these advancements. The incorporation of novel agents in the anti-lymphoma therapy will hopefully improve our current approach to relapsed FL patients, and eventually partially overcome the actual BMT indications.^37^ Nevertheless, until now, no novel drugs or combinations have shown a superior or non-inferior clinical outcome compared to the results of ASCT and alloSCT in relapsed/refractory FL patients. Unfortunately, transplant-based therapies are still affected by significant toxicities and for this reason, a carefully and multidisciplinary evaluation should always be provided to select eligible relapsed FL patients rightly.

## Figures and Tables

**Table 1:** Select outcomes of ASCT for relapsed or refractory FL.

	Number of Patients	R included in salvage CT	PFS	OS	Ref
**Schouten et al, 2000**	65	0	55–58% at 2 year	71–77% at 4 year	24
**Rohatiner et al, 2007**	121	0	55% at 5 year	71% at 5 years	44
**Sebban, et al, 2008**	98	33%	51% at 5 year	70% at 5 year	45
**Vose, et al, 2008**	248	Few (not reported)	44% at 5 year	63% at 5 year	46
**Tarella et al, 2008**	61+	100%	57 at 5 year	75% at 5 year	25
**Le Gouill et al, 2011**	112*	100%	52 at 3 year	92% at 3 year	43
**Le Gouill et al, 2011**	53*	0	40 at 3 year	63% at 3 year	43
**Evens et al, 2013**	135	100%	57% at 3 year	87% at 3 year	83
**Pettengell et al, 2013**	280	0	48–42% at 10 years	66.1–74.5% at 10 year	50
**Klyuchnikov et al**#**, 2015**	250	100%	41% at 5 year	74% at 5 year	41
**Klyuchnikov et al**$**, 2015**	136	100%	36% at 5 year	59 at 5 year	42

+ considered only R-HDS arm,

* Considering the trial end point, bot arm (with and without rituximab) are listed,

# Only Grade 2 FL were included in this study,

$ Only Grade 3 FL were included in this study.

**Table 2:** Novel published agents in FL in the last years.

New Agent	Classification	Reference	Cases	ORR	CR	PFS/TTP$
*Ibritumomab Tiuxetan*	Radio-conjugated MoAb&	Witzig et al. 2002^56^	54	74%	15%	Median 6.8 moths
*Ofatumumab*	MoAb anti-CD20	Czuczman, et al, 2012^63^	116	10–13%	0%	Median 5.8 moths
*Ibrutinib*	BTK inhibitor	Advani et al. 2012^58^	16	37%	18.5%	Median 13.6 months*
*Obinotuzumab*	MoAb anti-CD20	Sehn et al. 2014^65^	74	44.6%	12.2%	45.8% at 2 year
*Idelalisb*#	PI3Kδ inhibitor	Gopal et al, 2014^60^	72	57%	6%	Median 11 months
*R-Pidilizumab*	anti-PD MoAb	Westin et al. 2014^62^	32	66%	52%	Median 18.8 months
*Lenalidomide*	Immunomodulatory	Leonard et al, 2015^61^	45	53.3%	20%	27% at 2 year
*R-Lenalidomide*	Immunomodulatory	Leonard et al, 2015^61^	46	76.1%	39.1%	52% at 2 year
*Polatuzumab*+	Anti-CD79b drug conjugate MoAb	Palanca-Wessels et al, 2015^66^	16	43%	18.5%	Median 7.9 moths

$ PFS= progression free survival/TTP = time to progression,

& MoAb = monoclonal antibody,

* considering all lymphomas enrolled in the study,

# indolent lymphoma of whom 58% FL,

+ data related to indolent lymphoma study arm.

**Table 3:** A summary of outcomes of allo-SCT for relapsed or refractory FL underwent alloSCT.

Reference	n°pts	Conditioning regimen	TRM	EFS/PFS	OS	REF
Khouri, et al. 2001#	20	Flu/Cy - Flu/Cy/Ritux	10% at 2 year	84% at 2 year	84% at 2 year	79
Robinson et al. 2002	52	Fludarabine-based	22%	61% at 1 year	73% at 1 year	90
Morris et al. 2004%	41	Flu/Mel/Campath-1H	11% at 3 year	65% at 3 year	55% at 3 year	86
Faulkner et al. 2004&	28	BEAM/Campath-1H	13.3%	69% at 2 year	63.1% at 3 year	84
Corradini et al, 2007*	27	Flu/Cy/Thiotepa	14% at 3 year*	86% at 3 year	88% at 3 year	81
Khouri et al, 2008	47	Flu/Cy/Ritux	15% at 5 year	85% at 5 year	83% at 5 year	80
Hari et al, 2008	88	RIC	27% at 3 year	55% at 3 year	62% at 3 year	85
Hari et al, 2008	120	MAC	25% at 3 year	67% at 3 year	71% at 3 year	85
Thomson et al, 2010	82	Flu/Mel/Alemtuzumab	15% at 4 year	74% at 4 year	76% at 4 year	92
Pinana et al. 2010	37	Flu/Mel	41% at 4 year	57% at 4 year	54% at 4 year	87
Delgado et al. 2011	164	RIC	17% at 3 year	58% at 5 year	72% at 5 year	82
Robinson et al. 2013	149	RIC	22% at 3 year	57% at 5 year	67% at 5 year	89
Evens et al. 2013	48	RIC	24% at 3 year	52% at 3 year	61% at 3 year	83
Klyuchnikov et al. 2015	268	RIC	26% at 5 year	58% at 5 year	66% at 5 year	41
Klyuchnikov et al, 2016	61	RIC	27% at 5 year	51% at 5 year	54% at 5 year	42
Robinson 2016+	183	RIC	27% at 2 years	48% at 5 year	51% at 5 year	95

# Also Small Lymphocytic Lymphoma included,

% 29/41 of indolent lymphoma group were FL,

* including also other indolent lymphomas,

& including also other indolent lymphoma,

+ All patients relapsed after ASCT.
